# Erfolgreicher Wissensaustausch in virtuellen Teams – Wie wichtig ist soziale Präsenz?

**DOI:** 10.1007/s41449-021-00280-9

**Published:** 2021-10-13

**Authors:** Stephanie Tietz, Evi Kneisel, Katja Werner

**Affiliations:** 1 Fakultät Wirtschaftswissenschaften, Technische Universität Chemnitz, Thüringer Weg 7, 09126 Chemnitz, Deutschland; 2 Forschungsbereich Team- und Wissensmanagement, Technische Universität Chemnitz, Reichenhainer Straße 41, 09126 Chemnitz, Deutschland; 3 Studienbereich Wirtschaft, Berufsakademie Sachsen, Staatliche Studienakademie Glauchau, Kopernikusstraße 51, 08371 Glauchau, Deutschland

**Keywords:** Virtuelle Teams, Wissensaustausch, Soziale Präsenz, Virtual teams, Knowledge sharing, Social presence

## Abstract

Erfolgreicher Wissensaustausch ist ein zentraler Prozess für den Erfolg virtueller Teams. Damit gehen spezifische Herausforderungen einher. Die verwendete Informations- und Kommunikationstechnologien, die räumliche Trennung und der mangelnde persönliche Kontakt der Teammitglieder erschweren den Austausch von Wissen.

Die Forschung zu Einflussfaktoren auf Wissensaustausch in virtuellen Umgebungen fokussiert zumeist rein technische oder soziale Faktoren. Dagegen kaum beachtet ist die Rolle der sozialen Präsenz als interdisziplinäres Konstrukt. Soziale Präsenz meint das subjektive Erleben eines Zusammengehörigkeitsgefühls trotz physischer Distanz, das in der Interaktion zwischen den Nutzern und der vorhandenen Technologie entsteht.

Die vorliegende Studie untersuchte, inwieweit das Erleben sozialer Präsenz den Erfolg virtuellen Wissensaustausches beeinflusst. Basierend auf der Critical Incident Technique wurden 26 Interviews mit Mitgliedern virtueller Teams geführt. Die Teilnehmenden schilderten Situationen, in denen der virtuelle Wissensaustausch erfolgreich oder nicht erfolgreich war.

Die Auswertung zeigt, dass soziale Präsenz häufiger in erfolgreichen Situationen auftrat. Das weist darauf hin, dass deren Erleben wichtig für erfolgreichen Wissensaustausch ist.

*Praktische Relevanz:* Soziale Präsenz kann durch Medienreichhaltigkeit, Unterstützung sozialer Prozesse und Beziehungen in virtuellen Teams positiv beeinflusst werden. Aus den Ergebnissen lassen sich somit Implikationen für die Gestaltung, Moderation und Führung virtueller Zusammenarbeit ableiten.

## Abstract (en)

Effective knowledge sharing is a key process for the success of virtual teams. Several specific challenges like the information technology used, the geographical distribution and the lack of personal contact between team members make it difficult to share knowledge.

Current studies on influencing factors of effective knowledge sharing in virtual teams focus either mainly on technical or social factors. In contrast, little attention has been paid to the role of social presence as an interdisciplinary construct. Social presence means the individual or joint experience of a sense of togetherness despite physical distance, which arises in the interaction between the team members while using the existing technology.

The present study examines to what extent the experience of social presence influences the success of knowledge sharing in virtual teams.

Based on the Critical Incident Technique 26 interviews were conducted with members of virtual teams. The participants described situations in which the virtual knowledge exchange was successful or unsuccessful. The evaluation showed that social presence occurred more frequently in successful situations and emphasized its significance for a successful exchange of knowledge.

*Practical relevance: *Social presence can be positively influenced by media richness and support social processes and relationships in virtual teams. The results can be used to derive implications for virtual collaboration with the aim of optimizing knowledge sharing processes.

## Einleitung

### Hintergrund, Problemstellung und Zielsetzung

Mit zunehmender Internationalisierung bilden Unternehmen weltweite Netzwerke, in denen Teams organisationsübergreifend zusammenarbeiten. Dieser Trend wird durch die Entwicklung kollaborativer digitaler Technologien unterstützt. Neue Arbeitsformen entstehen. Sie ermöglichen es Unternehmen, orts- und zeitunabhängig Ressourcen zu bündeln und die Digitalisierung als Chance zur Weiterentwicklung wahrzunehmen (Killingsworth et al. 2016).

Bisher arbeiteten vor allem global agierende Organisationen in wissensintensiven und hochdynamischen Bereichen virtuell. Mit Beginn der COVID 19-Pandemie im Frühjahr 2020 und der damit verbundenen Umstellung auf das Arbeiten im Homeoffice sind jedoch derzeit nahezu alle Unternehmen von dieser Entwicklung betroffen, unabhängig von Branche, Größe, Bereich und Einsatzgebiet (Blanchard 2021; Klonek et al. 2021). So arbeiten 2021 ca. ein Viertel der Arbeitnehmer in Deutschland von zu Hause aus (Hans-Böckler-Stiftung 2021). Dies entspricht einer Zunahme von 20 % in den letzten zwei Jahren.

Virtuelle Teamarbeit ist somit zu einer bedeutenden Form der Arbeitsorganisation geworden, deren Erfolg die wirtschaftliche Entwicklung eines Unternehmens maßgeblich beeinflusst (Karl et al. 2021; Srivastava und Chandra 2018). Unter dem Begriff des virtuellen Teams wird in diesem Zusammenhang eine Gruppe von Personen verstanden, die über geografische, zeitliche und organisatorische Grenzen hinweg gemeinsam Aufgaben unter Nutzung von Informations- und Kommunikationstechnologien (IuK) löst (Piccoli et al. 2004; Zaglago et al. 2016). Da die zu bearbeitenden Problemstellungen in der Regel durch einen hohen Grad an Komplexität bestimmt sind, hängt der Erfolg virtueller Teams davon ab, ob es ihnen gelingt, das spezialisierte, verteilte Wissen ihrer Mitglieder zusammenzuführen (Fang 2017; Shah-Nelson et al. 2020). Die entscheidende Voraussetzung für diese Synthese ist ein erfolgreicher Austausch innerhalb des Teams als Basis für Problemlösungen und Innovationen (Bagherzadeh et al. 2019; Li et al. 2021).

Die fortschreitende Entwicklung der IuK offeriert eine Reihe virtueller Kollaborationswerkzeuge. Diese sorgen zum einen dafür, dass Wissen zwischen den Teammitgliedern dargestellt, vernetzt und transferiert werden kann. Zum anderen reduziert die räumliche Distanz jedoch die direkten und informellen Interaktionen zwischen den Teammitgliedern (Blanchard 2021). Letzteres beeinträchtigt den Aufbau von Vertrauen und den Zusammenhalt zwischen den Akteuren und wirkt negativ auf den Wissensaustausch (Aritz et al. 2018; Davidavičienė et al. 2020; Widjaja 2017).

Eine zentrale Bedeutung kommt dabei der Auswahl der eingesetzten IuK zu. So weisen Short et al. (1976) nach, dass die Wahl des Kommunikationsmediums die Fülle der übermittelten Informationen beeinflusst. Diese wiederum bestimmt die Interpretation der übertragenen Inhalte. Der Erfolg des Kommunikationsprozesses hängt somit vom Umfang der medialen Möglichkeiten bzw. der Medienreichhaltigkeit ab (Choi 2019; Yang et al. 2021). Dieses Phänomen wird dem Begriff der sozialen Präsenz zugeordnet (Short et al. 1976). Soziale Präsenz reduziert die Unsicherheit über die Gesprächsinhalte, stärkt das Vertrauen in das Gegenüber und bestimmt somit wesentlich den Erfolg der Kommunikation (Goggins et al. 2009; Kreijns et al. 2014). Diese Zusammenhänge werden in der Erforschung virtueller Zusammenarbeit aufgegriffen und bestätigt (Srivastava und Chandra 2018).

Dass der für Unternehmen wesentliche Erfolg des Wissensaustausches in hohem Maße von der Übermittlung und Interpretation des übertragenen Wissens abhängig ist, wurde ebenfalls nachgewiesen (Alavi und Leidner 2001; Singh 2020). Die Auswahl und Nutzung der verfügbaren Medien variieren jedoch mitunter stark. An dieser Stelle könnte das Konstrukt der sozialen Präsenz einen wesentlichen Beitrag zum Verständnis und zur Gestaltung erfolgreicher Wissensaustauschprozesse liefern. Bisher wurde die Rolle des Konstruktes vor allem in virtuellen Lernumgebungen untersucht, wie bspw. in Online-Diskussionsforen (Lowenthal und Dunlap 2020) und beim Online-Learning (Aldheleai und Tasir 2019; Whiteside et al. 2017). Die Befunde bestätigen, dass das Präsenzerleben in diesen Situationen einen positiven Einfluss auf Motivation, Engagement und das wahrgenommene Lernen hat (Aldheleai et al. 2020; Mitchell et al. 2021). Im Kontext virtueller Teamarbeit fand soziale Präsenz bislang kaum Beachtung. An dieses Forschungsdesiderat knüpft die vorliegende Studie an. Es wird die Bedeutung der sozialen Präsenz für den Erfolg von Wissensaustausch in virtuellen Teams untersucht. Daraus ergibt sich folgende Forschungsfrage:

„Welche Rolle spielt das Erleben von sozialer Präsenz für den Erfolg des Wissensaustausches in virtuellen Teams?“

Zur Beantwortung werden die beiden Forschungsbereiche Wissensaustausch und soziale Präsenz zusammengeführt. Nach der Definition grundlegender Begriffe und der Darstellung des Forschungsstandes erfolgt die Ableitung relevanter Kategorien sozialer Präsenz und die Formulierung der Hypothesen. Zur Testung dieser Annahmen werden 148 kritische Wissensaustauschsituationen in virtuellen Teams analysiert. Aufbauend auf einer quantitativen Inhaltsanalyse (Neuendorf 2017) wird geprüft, ob die erlebte soziale Präsenz häufiger in erfolgreichen als in nicht erfolgreichen Situationen erlebt wird. Die Ergebnisse lassen Rückschlüsse auf die Relevanz sozialer Präsenz für den Erfolg von Wissensaustausch zu. Unter Berücksichtigung der Limitationen der Studie können konkrete Handlungsimplikationen zur Förderung des Wissensaustausches in virtuellen Teams abgeleitet werden.

### Wissensaustausch in virtuellen Teams

Der Erfolg virtueller Teamarbeit hängt maßgeblich von der Befähigung der Teammitglieder ab, das vorhandene, zumeist verteilte, Fach- und Erfahrungswissen für gemeinsame Problemlösungen und Entscheidungsprozesse zu nutzen (Fang et al. 2014; Shah-Nelson et al. 2020; Singh 2020). Dabei bilden der kontinuierliche Austausch sowie die Integration heterogenen Wissens die Grundlage für kollektive und organisationale Lernprozesse und Innovationen (Arfi et al. 2020; Edmondson 2002; Yeo 2020).

Wissensaustausch ist ein aktiver und dynamischer Prozess, der auf zwischenmenschlichen Interaktionsprozessen basiert, bspw. im Rahmen von Diskussionen und gemeinsamen Problemlöseprozessen (Söderquist 2006). Er zielt auf eine gegenseitige Beeinflussung von Erfahrungen und Wissen ab (Ipe 2003). Die mit der Abhängigkeit von technischen Medien und der physischen Distanz virtueller Teams verbundene Abnahme direkter persönlicher Interaktionen schränkt die Qualität und Häufigkeit von Wissensaustauschprozessen ein (Olaisen und Revang 2017). Die Kommunikations- und Interaktionsprozesse in virtuellen Teams werden fehleranfälliger. So führt die Absenz von Körpersprache häufig zu Fehlinterpretationen, was wiederum den Erfolg der Zusammenarbeit negativ beeinflusst (Beldarrain und Diehl 2019; Klitmøller und Lauring 2013). Ferner ist die Identifikation individueller Fachkenntnisse schwierig und damit deutlich zeitaufwendiger als in Präsenzformaten (Kauppila et al. 2011; Koste und Haerem 2016). IuK werden zudem häufig als unpersönlich empfunden, da nonverbale und beziehungsbezogene Hinweise fehlen (Blanchard 2021; Walther und Parks 2002). Dieser Mangel und die geringere Kontakthäufigkeit in virtuellen Kontexten schränkt den Aufbau sozialer Verbindungen ein (Yilmaz 2017).

Untersuchungen zu virtuellem Wissensaustausch stellen die Bedeutung persönlicher Begegnungen, kohärenter sozialer Bindungen, gemeinsamer Normen und Vertrauen für den Erfolg von Wissensaustausch in virtuellen Teams heraus (Kauppila et al. 2011; Lucas 2006; Michailova und Minbaeva 2012). In diesem Zusammenhang spielt das Konstrukt der sozialen Präsenz eine wesentliche Rolle, da es die Basis für gegenseitiges Vertrauen und soziale Verbundenheit schafft (Lowenthal und Mulder 2017; Short et al. 1976).

### Soziale Präsenz und Wissensaustausch

#### Definition und Dimensionen sozialer Präsenz

Soziale Präsenz beschreibt das bewusste Erleben von Beteiligung und sozialer Interaktion zwischen Mitgliedern kollaborativer Gruppen (Goggins et al. 2009; Koh et al. 2007; Picciano 2002). Ein soziales Präsenzerleben, auch in der mobilen Zusammenarbeit, hat einen positiven Einfluss auf die Interaktions‑, Partizipations- und Kollaborationsbereitschaft (Kreijns et al. 2014; Yilmaz 2017) als wichtige Voraussetzungen für Wissensaustauschprozesse. Im virtuellen Raum ermöglicht die soziale Präsenz die subjektive Wahrnehmung von Zusammensein sowie das Erleben sozialer Verbundenheit (Lowenthal und Snelson 2017; Yilmaz 2017). Es ist somit davon auszugehen, dass das Erleben sozialer Präsenz im Team dazu beitragen kann, die negativen Folgen räumlicher Distanz zu überwinden und erfolgreichen Wissensaustausch zu unterstützen.

Obwohl soziale Präsenz häufig intuitiv als das Gefühl definiert wird, in einer virtuellen Umwelt präsent zu sein, existieren verschiedene Variationen des Terminus und seiner Konzeptualisierung (Garrett et al. 2017; Kreijns et al. 2014; Lowenthal und Snelson 2017). So ist es oft schwer zu unterscheiden, ob soziale Interaktion, Unmittelbarkeit, Intimität oder Verbundenheit gemeint ist, wenn der Begriff soziale Präsenz verwendet wird (Lowenthal 2010). Ursache dafür ist, dass verschiedene Forschungsbereiche und theoretische Strömungen sich mit sozialer Präsenz auseinandersetzen und die Arbeiten auf unterschiedlichen theoretischen Schwerpunkten basieren (Lowenthal und Snelson 2017; Rüggenberg 2007). Um der Begriffsvielfalt zu begegnen und eine geeignete Operationalisierung sozialer Präsenz in Verbindung mit Wissensaustausch in virtuellen Teams zu finden, ist ein Rückblick auf die Ursprünge des Konstruktes sinnvoll.

Vorreiter der Theorie sozialer Präsenz waren Short et al. (1976). Ihr Anliegen war, zwischenmenschliche Auswirkungen bei der Nutzung verschiedener Kommunikationsmedien (z. B. Videokanäle, Telefon und persönliche Treffen) im organisationalen Kontext zu erklären. Unter sozialer Präsenz verstehen sie das Ausmaß der bewussten Wahrnehmung anderer Personen in der digitalen Zusammenarbeit und das sich anschließende Bewusstsein einer persönlichen Beziehung zu diesen Personen. Diese Wahrnehmung wird nach den Autoren in erster Linie durch das verwendete Medium bestimmt und ist daher hauptsächlich technologiebasiert (Short et al. 1976). Weiterführende Konzeptualisierungen betonen neben der technologischen Komponente die Bedeutung sozialer Kontingenzfaktoren für das Erleben sozialer Präsenz. So betont Heeter (1992), dass soziale Präsenz in erster Linie das Gefühl des Zusammenseins („being together“) widerspiegelt und nicht nur das des bloßen Daseins („being there“). Biocca et al. (2003) gehen in ihrem Theorieentwurf ebenfalls davon aus, dass soziale Präsenz ein Merkmal der interagierenden Personen ist und keine reine Eigenschaft des technischen Mediums: „[...] social presence cannot really be conceptualized as fixed property of medium. Rather it is best conceptualized as a property of individual perceptions of mediated others, that likely fluxates during interactions, tasks, and individual differences“ (Biocca et al. 2003, S. 30). Kreijns et al. (2014) kommen schließlich zu dem Ergebnis, dass soziale Präsenz sowohl durch die physischen Eigenschaften der Kommunikationsmedien als auch durch soziale Faktoren, wie interpersonelle Beziehungen, Kohäsion und Privatheit, bestimmt wird. Das heißt, dass selbst mit rein textbasierten, asynchronen IuK ein hohes Maß an sozialer Präsenz erreicht werden kann, z. B. durch soziale Verbindungen zwischen den Gruppenmitgliedern (Lowenthal und Mulder 2017).

In Anlehnung an diese Vorarbeiten werden im Folgenden sowohl technologische als auch soziale Faktoren berücksichtigt, um soziales Präsenzerleben adäquat zu bestimmen. Dabei wird differenziert zwischen einer *technologiebasierten Präsenzwahrnehmung*, die durch die Eigenschaften des Mediums bestimmt wird (Lowenthal und Snelson 2017; Short et al. 1976), und einer *beziehungsbasierten Präsenzwahrnehmung,* die durch den sozialen Kontext bestimmt wird (Biocca et al. 2003; Kreijns et al. 2014). Beide werden als relevante Dimensionen des Erlebens sozialer Präsenz herangezogen und in Beziehung zu erfolgreichen Wissensaustausch in virtuellen Teams gesetzt.

#### Technologiebasierte soziale Präsenz und Wissensaustausch

Die Dimension der *technologiebasierten sozialen Präsenz *bezieht sich auf die Wahrnehmung, in einer virtuellen Umgebung mit realen Personen zu interagieren und selbst Teil dieser Realität zu sein. Grundlage hierfür bildet die empfundene Qualität des Mediums und ob dieses in der Lage ist, ein Gefühl der sozialen Präsenz zu unterstützen. Diese Beurteilung ist subjektiver Natur, erwächst jedoch aus den objektiv bestehenden Eigenschaften des Mediums, z. B. dessen Synchronizität. Im Folgenden werden in Anlehnung an die Originalkonzeption von Short et al. (1976) und darauf aufbauender Forschungsarbeiten (Heeter 1992; Kreijns et al. 2014; Lowenthal und Snelson 2017; Tu und McIsaac 2002) drei Kategorien für das Erleben technologiebasierter sozialer Präsenz abgeleitet und ihr Einfluss auf Wissensaustausch begründet: 1a Wahrnehmung anderer Teammitglieder als reale Personen, 1b Wahrnehmung der eigenen Person als realen Teil des virtuellen Raums und 1c Unmittelbarkeit der Interaktion.

Die erste Kategorie, die *1a Wahrnehmung anderer Teammitglieder als reale Person, *beschreibt das Ausmaß des empfundenen Realsein anderer Personen (Kreijns et al. 2014). Das zweite Kriterium, die* 1b Wahrnehmung der eigenen Person als realen Teil des virtuellen Raums, *bezieht sich hingegen auf das Gefühl, selbst Teil der virtuellen Kommunikation zu sein und von anderen Teilnehmern als real wahrgenommen zu werden (Garrison et al. 2010). Beide Kriterien werden maßgeblich durch die Eigenschaften der verwendeten IuK bestimmt (Short et al. 1976). Durch die Verwendung reichhaltiger Medien (z. B. Telepresence-Systeme) können verbale, nonverbale und sozio-emotionale Signale und Reaktionen (Feedback) gesendet und empfangen werden, was wiederum die Realitätswahrnehmung unterstützt (Allmendinger 2010; Fairchild et al. 2017). Ein hoher Realitätsgrad, sowohl bezogen auf andere als auch auf die eigene Person, fördert die Bereitschaft zur aktiven Teilnahme am virtuellen Austausch (Kreijns et al. 2014; Oh et al. 2018). Partizipation und Interaktionsbereitschaft sind wiederum relevante Voraussetzungen für das Gelingen des Wissensaustausches (Schofield et al. 2018; Shouhong und Wang 2018). Entsprechend wird davon ausgegangen, dass beide Kriterien, die Realitätswahrnehmung anderer Teammitglieder und die der eigenen Person, den Erfolg des Wissensaustauschs in virtuellen Teams positiv beeinflussen.

*Hypothese 1a: *In erfolgreichen Wissensaustauschsituationen werden andere Teammitglieder häufiger als reale Personen wahrgenommen als in nicht erfolgreichen Wissensaustauschsituationen.

*Hypothese 1b: *In erfolgreichen Wissensaustauschsituationen nehmen sich Teammitglieder häufiger als realen Teil des virtuellen Raums wahr als in nicht erfolgreichen Wissensaustauschsituationen.

Die dritte Kategorie *1c Unmittelbarkeit *bezieht sich auf die Direktheit des virtuellen Austausches. Kennzeichen einer hohen Unmittelbarkeit sind geringe zeitliche Verzögerungen in der Kommunikation und die Möglichkeit sich schnell, unvermittelt und informell auszutauschen (Brown et al. 2010; Dahlstrom-Hakki et al. 2020). Eine hohe Unmittelbarkeit wird insbesondere in synchronen Austauschsituationen erlebt, beispielsweise via Chat oder Telefon. Erste Befunde weisen darauf hin, dass unmittelbarer Austausch aufgabenbezogene Konflikte und Unsicherheiten reduziert (Kankanhalli et al. 2006) sowie Engagement und den Lernerfolg positiv beeinflusst (Dixson et al. 2017). Im Folgenden wird angenommen, dass Unmittelbarkeit positiv auf den Erfolg virtuellen Wissensaustausches wirkt, da unmittelbare Reaktionen und Feedback das Kommunikationsverständnis verbessern (Brown et al. 2010).

*Hypothese 1c: *In erfolgreichen Wissensaustauschsituationen werden Interaktionen häufiger als unmittelbar wahrgenommen als in nicht erfolgreichen Wissensaustauschsituationen.

#### Beziehungsbasierte soziale Präsenz und Wissensaustausch

Die Dimension der *beziehungsbasierten sozialen Präsenz* wird durch Merkmale der sozialen Verbindung zwischen den Teammitgliedern bestimmt. Kennzeichen sind die Wahrnehmung von Verbundenheit und zwischenmenschlichen Beziehungen zu anderen Teammitgliedern. Bei hohem beziehungsbasierten Präsenzerleben entsteht ein gemeinsamer sozialer Raum als Grundlage für soziale Interaktionen und Wissensaustausch Kreijns et al. (2014), Díaz et al. (2010), Kreijns et al. (2014) sowie Tu und McIsaac (2002) werden im Folgenden vier Kriterien herangezogen, um das Erleben beziehungsbasierter sozialer Präsenz in virtuellen Teams zu beschreiben und in Beziehung zu erfolgreichen Wissensaustausch zu setzen: 2a gegenseitige Vertrautheit, 2b Teamkohäsion, 2c sozialer Komfort und 2d ein gemeinsames Verständnis.

Der Aspekt der* 2a gegenseitigen Vertrautheit *beschreibt das Gefühl der Teammitglieder sich persönlich nah zu sein und gut zu kennen. Merkmale von Vertrautheit sind die Wahrnehmung einer sozialen Beziehung zur anderen Person, die Verwendung einer informellen und affektiv geprägten Sprache sowie Intimität im Umgang miteinander (Tu und McIsaac 2002). Gegenseitige Vertrautheit fördert Sicherheit und Verbundenheit (Eisenberg und Mattarelli 2017) und wirkt sich positiv auf die eigene Kompetenzwahrnehmung aus (Deci und Ryan 2000). Yoon und Rolland (2012) bestätigen, dass diese Faktoren die individuelle Bereitschaft steigern, eigenes Wissen aktiv einzubringen. Entsprechend wird angenommen, dass die Wahrnehmung von Vertrautheit in virtuellen Teams einen positiven Einfluss auf den Wissensaustausch hat.

*Hypothese 2a: *In erfolgreichen Wissensaustauschsituationen wird gegenseitige Vertrautheit häufiger wahrgenommen als in nicht erfolgreichen Wissensaustauschsituationen.

Das zweite Kriterium *2b Teamkohäsion *bezieht sich auf ein hohes Maß an Teamzusammenhalt und ein ausgeprägtes Gemeinschaftsgefühl (Díaz et al. 2010). In kohäsiven Teams erleben die Mitglieder ein starkes Zugehörigkeits- und Identifikationsgefühl (Wir-Gefühl) mit der Gruppe. Sie unterstützen sich gegenseitig, nehmen an Gruppenaktivitäten teil und betrachten Gruppenziele als ihre eigenen, was sich nachweislich positiv auf die Leistungsfähigkeit auswirkt (Beal et al. 2003). Für Präsenzteams wurde nachgewiesen, dass ein positiver Zusammenhang zwischen Kohäsion und der Bereitschaft zum Wissensaustausch besteht (Kakar 2018; Reagans und McEvily 2003). In Folge wird angenommen, dass Kohäsion auch in virtuellen Teams einen positiven Effekt auf Wissensaustausch hat, da sie die Kommunikation, Koordination und Zusammenarbeit zwischen den Teammitgliedern verbessert.

*Hypothese 2b: *In erfolgreichen Wissensaustauschsituationen wird Teamkohäsion häufiger wahrgenommen als in nicht erfolgreichen Wissensaustauschsituationen.

*2c Sozialer Komfort *als dritte Kategorie beziehungsbasierten sozialen Präsenzerlebens wird von Carlon et al. (2012) als das Erleben einer positiven Grundstimmung im Team beschrieben. Kennzeichen sind die Wahrnehmung einer offenen und vertrauensvollen Umgebung sowie das Erleben von Spaß und Freude in der Zusammenarbeit. Eine positive Atmosphäre im Team, geprägt von Vertrauen und psychologischer Sicherheit, fördert das Engagement der Teammitglieder untereinander und verringert die Angst ausgenutzt zu werden (Chow und Chan 2008). Dies sollte sich positiv auf die Bereitschaft zur wechselseitigen Interaktion und Wissensaustausch auswirken.

*Hypothese 2c: *In erfolgreichen Wissensaustauschsituationen wird sozialer Komfort häufiger wahrgenommen als in nicht erfolgreichen Wissensaustauschsituationen.

Die vierte Kategorie, die *2d Wahrnehmung eines gemeinsamen Verständnisses, *bezieht sich auf die erlebte Übereinstimmung in arbeitsbezogenen Auffassungen, Einstellungen, Werten und Perspektiven hinsichtlich wichtiger Aspekte der Zusammenarbeit (Bittner und Leimeister 2014; Mulder und Swaak 2002). Ein gemeinsames Verständnis ergibt sich durch den Wechsel von individuellen Perspektiven zu einer gemeinsamen Perspektive, die von der Gruppe akzeptiert wird. Dieses fördert die Partizipation der Teammitglieder an der gemeinsamen Aufgabenbearbeitung (Miles und Kivlighan 2008), reduziert das Risiko von Aufgabenkonflikten (Tjosvold 1998) und verringert Missverständnisse und Meinungsverschiedenheiten (Jackson et al. 1995; Kellermann et al. 2008; Rouse et al. 1992), die in positivem Zusammenhang zu Wissensaustausch stehen (Rosenkranz et al. 2014).

*Hypothese 2d: *In erfolgreichen Wissensaustauschsituationen wird ein gemeinsames Verständnis häufiger wahrgenommen als in nicht erfolgreichen Wissensaustauschsituationen.

## Methodik

### Datengrundlage und Stichprobe

Die Überprüfung der Hypothesen erfolgte anhand der Ergebnisse einer Interviewstudie in acht mittelständischen deutschen Unternehmen, die in ihren Kernprozessen unter Einsatz virtueller Teamarbeit agieren. Alle Firmen stammen aus der IT-Branche mit Schwerpunkt auf Software-Entwicklung und Beratung.

Die Stichprobe umfasste 26 Fach- und Führungskräfte virtueller Teams. Zum Erhebungszeitpunkt arbeiteten die Teilnehmenden mindestens drei Jahre in virtuellen Gruppen und mindestens sechs Monate im gegenwärtigen Team. Sie verfügten somit über umfassende und aktuelle Erfahrungen mit virtuellen Wissensaustauschprozessen. Alle Befragten haben einen Universitätsabschluss oder eine einschlägige Berufsausbildung und arbeiteten zum Zeitpunkt der Erhebung in funktionsübergreifenden Positionen, u. a. in Führungs‑, Beratungs- und Entwicklungsfunktionen. 19 der 26 Befragten (73 %) waren männlich und 7 (27 %) weiblich. Ihr Alter lag zwischen 24 und 55 Jahren mit einem Durchschnittsalter von 35 Jahren (SD = 9,06). Die durchschnittliche Mitgliedszeit im aktuellen Team betrug 2,8 Jahre (SD = 1,94).

### Datenerhebung

Die Datenerhebung basierte auf der Methode der kritischen Ereignisse (Critical Incident Technique, kurz: CIT) von Flanagan (1954). Das allgemeine Ziel der CIT besteht darin, Verhaltensweisen zu sammeln, die bei positiven oder negativen Arbeitssituationen erfolgskritisch sind (Flanagan, 1954). Ursprünglich wurde die CIT zur Arbeitsplatzanalyse und zur Identifizierung von Kriterien entwickelt, die für eine erfolgreiche Arbeitsleistung entscheidend sind. Später wurde die CIT erfolgreich auf weitere organisationsbezogene Fragestellungen übertragen, bspw. Entscheidungsprozesse (Coetzer und Redmond 2011; Francis-Smythe et al. 2013; MacDonald et al. 2008), Führungserfolg (Hamlin und Patel 2017; Lekchiri et al. 2018) und teambezogene Kompetenzen (Irwin et al. 2016).

In der vorliegenden Studie stand die Erforschung erfolgreicher Wissensaustauschprozesse in virtuellen Teams im Fokus. Hierfür sollten die Teilnehmenden kritische Ereignisse beschreiben, in denen der Wissensaustausch besonders erfolgreich oder nicht erfolgreich verlaufen ist. Im Rahmen der Interviews wurden Erfolg und Misserfolg als zwei gegensätzliche Pole einer Dimension betrachtet, die anhand konkreter Ergebnisse des Wissensaustauschs bewertet werden können. Die Teilnehmenden beschrieben die Situationen virtueller Zusammenarbeit so konkret und umfassend wie möglich, inklusive Anlass, Verlauf und beteiligte Personen. Da die vorliegende Studie auf die Erfassung des Erlebens sozialer Präsenz abzielte, wurde der ursprüngliche Verhaltensfokus der CIT um kognitive und affektive Faktoren erweitert.

Die Interviews waren halbstrukturiert. Zwei Interviewerinnen folgten einem festgelegten Leitfaden und stellten dieselben offenen Fragen. Ein Pretest mit zwei Interviewpartnern diente der Testung des Verständnisses der Leitfragen und lieferte gleichzeitig die Grundlage für vertiefende Fragen zur detaillierten Beschreibungen. Ziel war eine möglichst konkrete Beschreibung der Ereignisse durch die Befragten. Die Schilderungen sollten das Gefühl auslösen, die Situation selbst beobachtet zu haben (Flanagan 1954). Die Interviews dauerten zwischen 30 und 60 min. Nach Erläuterung des allgemeinen Ziels der Studie wurden die Interviewpartner darauf hingewiesen, dass die Interviews streng vertraulich seien und ihre Namen nicht veröffentlicht würden. Zusätzlich wurden die Teilnehmenden um ihre Erlaubnis gebeten, die Interviews aufzuzeichnen. Alle Interviewpartner gaben ihr Einverständnis.

Nach der allgemeinen Einführung wurden die Interviewees gebeten: *„Denken Sie an eine Situation in der Zusammenarbeit in Ihrem Team, in der ein erfolgreicher oder nicht erfolgreicher Wissensaustausch stattgefunden hat. Bitte versuchen Sie sich an das konkrete Verhalten der Teammitglieder und die Interaktionen zwischen den Teammitgliedern zu erinnern, die zu dem (nicht) erfolgreichen Wissensaustausch geführt haben. Beschreiben Sie diese Situation und das Verhalten der Teammitglieder möglichst genau.“*

Um weitere Informationen über die jeweilige Situation zu erhalten, wurden nacheinander die folgenden Fragen gestellt: *Welche allgemeinen Umstände führten zu diesem Ereignis? Welche Teammitglieder waren beteiligt? Wie hat sich die Situation entwickelt? Bitte erläutern Sie möglichst genau die Situation und wie sie diese erlebt haben. Was haben die Teammitglieder getan, damit der Austausch erfolgreich oder nicht erfolgreich verlief? Welche Konsequenzen hatte dieses Ereignis?*

Im Anschluss an die Beschreibung der kritischen Ereignisse wurden die Teilnehmenden gebeten, das Ergebnis des Wissensaustauschs konkret zu benennen. Dies diente der Kontrolle der Interviewinstruktion. Abschluss der Interviews bildeten demografische Fragen zu Alter und beruflichem Hintergrund.

### Datenanalyse und -auswertung

Insgesamt wurden ca. 1200 min Datenmaterial erhoben, transkribiert und mittels der Softwares MAXQDA Analytics Pro 2020 und SPSS 27 ausgewertet.

Für die Analyse des Auftretens sozialer Präsenz wurde eine quantitative Inhaltsanalyse (Neuendorf 2017) durchgeführt. Dafür wurde der Ansatz der strukturierenden Inhaltsanalyse nach Mayring und Fenzl (2014) genutzt, welcher ein systematisches Verfahren zur deduktiven Kodierung qualitativer Daten bietet.

In einem ersten Schritt wurden die transkribierten Interviews in eine Liste kritischer Ereignisse umgewandelt, die übergreifende Analyseeinheiten darstellen. Im zweiten Schritt kodierten zwei wissenschaftliche Mitarbeiterinnen unabhängig voneinander jeden Incident hinsichtlich des Auftretens der sozialen Präsenz. Grundlage bildete ein standardisiertes Kategoriensystem inklusive Kodierleitfaden. Das Kodiersystem basierte auf den theoretisch abgeleiteten Dimensionen sozialer Präsenz (vgl. Abschn. 1.3.2 und 1.3.3) und beinhaltete die folgenden sieben Kategorien inklusive Kodierregeln: 1a Wahrnehmung anderer Teammitglieder als reale Person, 1b Wahrnehmung der eigenen Person als realen Teil des virtuellen Raums, 1c Unmittelbarkeit, 2a gegenseitige Vertrautheit, 2b Teamkohäsion, 2c sozialer Komfort und 2d gemeinsames Verständnis. Die resultierende durchschnittliche Interrater-Reliabilität war zufriedenstellend (Cohens Kappa = 0,81). Alle Abweichungen wurden diskutiert bis ein Konsens erzielt wurde. Im dritten Schritt wurden für jede Kategorie Auftretenshäufigkeiten berechnet und die Anzahl der kritischen Situationen bestimmt, in denen diese Kategorie vorkam. Um das Auftreten der sozialen Präsenzkriterien über alle erfolgreichen und nicht erfolgreichen Wissensaustauschsituationen zu vergleichen, wurden Chi-Quadrat-Tests durchgeführt. Die Bewertung der Effektstärken erfolgt in Anlehnung an Cohen (1988).

## Ergebnisse

Die Interviewpartner schilderten jeweils zwei bis elf kritische Ereignisse zum Wissensaustausch in virtuellen Teams (M = 5,77, SD = 2,34). Insgesamt wurden von allen Teilnehmenden 148 kritische Ereignisse berichtet. Bei diesen handelte es sich um 90 (61 %) erfolgreiche und 58 (39 %) nicht erfolgreiche Wissensaustauschsituationen. Die Kategorien sozialer Präsenz konnten in insgesamt 92 der beschriebenen Situationen nachgewiesen werden. Dabei handelte es sich um 68 (74 %) erfolgreiche Situationen und 24 (26 %) nicht erfolgreiche Situationen.

Ein Chi-Quadrat-Test zur Analyse der Verteilung der Ereignisse, bezogen auf deren Bewertung (erfolgreich vs. nicht erfolgreich) und dem Auftreten sozialer Präsenz (nachgewiesen vs. nicht nachgewiesen), zeigt eine signifikante Ungleichverteilung (Χ^2^ (1) = 17,51, *p* = 0,006). Wie in Tab. 1 ersichtlich wird, wurde das Erleben sozialer Präsenz insgesamt häufiger in erfolgreichen Wissensaustauschsituationen berichtet als in nicht erfolgreichen Situationen.

**Table 1:** 

Auftreten sozialer Präsenz	Situationstyp		
	Erfolgreicher Wissensaustausch	Nicht erfolgreicher Wissensaustausch	Summe
Soziale Präsenz nachgewiesen	68	24	92
Soziale Präsenz nicht nachgewiesen	22	34	56
Kritische Ereignisse gesamt	90	58	148

Für eine differenzierte Analyse des Auftretens sozialer Präsenz erfolgte für jede der sieben Kategorien sozialer Präsenz (1a Wahrnehmung anderer Teammitglieder als reale Person, 1b Wahrnehmung der eigenen Person als realen Teil des virtuellen Raums, 1c Unmittelbarkeit, 2a gegenseitige Vertrautheit, 2b Teamkohäsion, 2c sozialer Komfort und 2d gemeinsames Verständnis) die Bestimmung der Auftretenshäufigkeit in den kritischen Ereignissen. Dabei wurde zwischen erfolgreichen und nicht erfolgreichen Wissensaustauschsituationen unterschieden. In Tab. 2 sind die Ergebnisse dargestellt.

Die deskriptive Analyse zeigt, dass alle sieben Kategorien in den Daten nachgewiesen werden konnten. Zudem wurden die Kategorien sowohl in erfolgreichen als auch nicht erfolgreichen Situationen genannt. In den meisten Situationen konnte die Kategorie *1a Wahrnehmung anderer Teammitglieder als reale Person *(*n* = 48, 32 %) nachgewiesen werden, gefolgt von den vier Kategorien* 2b Teamkohäsion *in 41 (28 %) Situationen, *2c sozialer Komfort* in 38 (26 %) Situationen, *2d gemeinsames Verständnis* in 28 (19 %) Situationen und *2a Vertrautheit* in 25 (17 %) Situationen. Die zwei Kriterien *1b Wahrnehmung der eigenen Person als realen Teil des virtuellen Raums *und *1c Unmittelbarkeit* konnten dagegen nur in 21 (14 %) bzw. 11 (7 %) der kritischen Ereignisse kodiert werden.

**Table 2:** 

Kategorien sozialer Präsenz	Gesamtanzahl (%) kritischer Ereignisse, in denen die Kategorie nachgewiesen wurde	Anzahl (%) erfolgreicher Ereignisse, in denen die Kategorie nachgewiesen wurde	Anzahl (%) nicht erfolgreicher Ereignisse, in denen die Kategorie nachgewiesen wurde	Chi-Quadrat-Verteilung			
				Chi (Χ^2^)	*p* von Chi (Χ^2^)	Cramer	Phi (Φ)
*Dimension 1: Technologiebasierte soziale Präsenz*							
1a Wahrnehmung anderer Teammitglieder als reale Person	48 (32 %)	39 (43 %)	9 (16 %)	12,453	0,000	0,290	0,290
1b Wahrnehmung der eigenen Person als realen Teil des virtuellen Raums	21 (14 %)	17 (19 %)	4 (7 %)	4,166	0,041	0,168	0,168
1c Unmittelbarkeit	11 (7 %)	8 (9 %)	3 (5 %)	0,708	0,400	0,069	0,069
*Dimension 2: Beziehungsbasierte soziale Präsenz*							
2a Vertrautheit	25 (17 %)	21 (23 %)	4 (7 %)	6,788	0,009	0,214	0,214
2b Teamkohäsion	41 (28 %)	35 (39 %)	6 (10 %)	14,348	0,000	0,311	0,311
2c sozialer Komfort	38 (26 %)	31 (34 %)	7 (12 %)	9,253	0,002	0,250	0,250
2d gemeinsames Verständnis	28 (19 %)	20 (22 %)	8 (14 %)	1,634	0,201	0,105	0,105

Zur Überprüfung der Hypothesen (Tab. 3) wurden Chi-Quadrat-Verteilungstests durchgeführt. Für jede der sieben Kategorien wurde geprüft, inwieweit es signifikante Unterschiede in deren Verteilung in erfolgreich bzw. nicht erfolgreichen gab.

Eine differenzierte Analyse anhand der *p*-Werte in Tab. 2 zeigt, dass die zwei technologiebasierten Kategorien *1a Wahrnehmung anderer Teammitglieder als reale Person *(Χ^2^(1) = 12,453; Φ = 0,290; *p* = 0,000) und die *1b Wahrnehmung der eigenen Person als realen Teil des virtuellen Raums *(Χ^2^(1) = 4,166; Φ = 0,168, *p* = 0,041) signifikant häufiger in erfolgreichen Ereignissen auftraten. Für die dritte Kategorie *1c Unmittelbarkeit* wurde kein signifikanter Unterschied festgestellt (Χ^2^(1) = 0,708; Φ = 0,069; *p* = 0,400). Somit können die *Hypothesen 1a* und *1b* bestätigt werden: In erfolgreichen Wissensaustauschsituationen werden andere Teammitglieder sowie die eigene Person häufiger als reale Personen erlebt. *Hypothese 1c* muss abgelehnt werden: In erfolgreichen Wissensaustauschsituationen wird die Unmittelbarkeit der Interaktion nicht häufiger berichtet.

Des Weiteren zeigen die Ergebnisse, dass die drei Kategorien beziehungsbasierter sozialer Präsenz *2a Vertrautheit* (Χ^2^(1) = 6,788; Φ = 0,214; *p* = 0,009), *2b Teamkohäsion* (Χ^2^(1) = 14,348; Φ = 0,311; *p* = 0,000) und *2c sozialer Komfort* (Χ^2^(1) = 9,253; Φ = 0,250; *p* = 0,002) signifikant häufiger in erfolgreichen Ereignissen wahrgenommen wurden im Vergleich zu nicht erfolgreichen Ereignissen. Für die Kategorie *2d gemeinsames Verständnis* zeigte sich dagegen kein signifikanter Unterschied (Χ^2^(1) = 1,634; Φ = 0,105; *p* = 0,201). In Folge können die *Hypothesen 2a, 2b* und *2c* bestätigt werden: In erfolgreichen Wissensaustauschsituationen haben die Teilnehmenden häufiger gegenseitige Vertrautheit, Teamkohäsion und sozialen Komfort wahrgenommen. *Hypothese 2d* muss abgelehnt werden: Ein gemeinsames Verständnis wurde in erfolgreichen Austauschsituationen nicht signifikant häufiger wahrgenommen.

Die nachfolgende Tab. 3 beinhaltet einen Überblick über die Ergebnisse der Hypothesentests.

**Table 3:** 

Nr	Hypothesen	Befund
*Dimension 1: technologiebasierte soziale Präsenz*		
1a	In erfolgreichen Wissensaustauschsituationen werden andere Teammitglieder häufiger als reale Personen wahrgenommen als in nicht erfolgreichen Wissensaustauschsituationen	Bestätigt
1b	In erfolgreichen Wissensaustauschsituationen nehmen sich Teammitglieder häufiger als realen Teil des virtuellen Raums wahr als in nicht erfolgreichen Wissensaustauschsituationen	Bestätigt
1c	In erfolgreichen Wissensaustauschsituationen werden Interaktionen häufiger als unmittelbar wahrgenommen als in nicht erfolgreichen Wissensaustauschsituationen	Nicht bestätigt
*Dimension 2: beziehungsbasierte soziale Präsenz*		
2a	In erfolgreichen Wissensaustauschsituationen wird gegenseitige Vertrautheit häufiger wahrgenommen als in nicht erfolgreichen Wissensaustauschsituationen	Bestätigt
2b	In erfolgreichen Wissensaustauschsituationen wird Teamkohäsion häufiger wahrgenommen als in nicht erfolgreichen Wissensaustauschsituationen	Bestätigt
2c	In erfolgreichen Wissensaustauschsituationen wird sozialer Komfort häufiger wahrgenommen als in nicht erfolgreichen Wissensaustauschsituationen	Bestätigt
2d	In erfolgreichen Wissensaustauschsituationen wird ein gemeinsames Verständnis häufiger wahrgenommen als in nicht erfolgreichen Wissensaustauschsituationen	Nicht bestätigt

## Diskussion

Die Relevanz von Wissensaustausch für die erfolgreiche Zusammenarbeit in virtuellen Teams wird sowohl in der Forschung (Fang et al. 2014) als auch in der organisationalen Praxis immer deutlicher hervorgehoben. Insbesondere die Frage, wie Wissensaustausch in virtuellen Teams gefördert werden kann, ist in den letzten Jahren in den Fokus gerückt (Shah-Nelson et al. 2020). Einen wesentlichen Beitrag zur Unterstützung der Wissensübertragung und -vernetzung in E‑Learningformaten leistet die soziale Präsenz (Yilmaz 2017). Es steht zu vermuten, dass diese Ergebnisse auf virtuelle Teamarbeit in Unternehmen übertragbar sind. Die vorliegende Studie setzt an diesem Forschungsdesiderat an und untersucht die Bedeutung sozialer Präsenz für erfolgreichen Wissensaustausch. Die Forschungsfrage lautet: Welche Rolle spielt das Erleben von sozialer Präsenz für den Erfolg des Wissensaustausches in virtuellen Teams? Auf Grundlage einer umfassenden Kategorisierung theoretischer Konzepte und empirischer Befunde wurden für die zwei Dimensionen technologiebasierte und beziehungsbasierte soziale Präsenz und sieben Kategorien abgeleitet und ihre Bedeutung in kritischen Wissensaustausch Situationen analysiert. Dabei handelt es sich um einen ersten Beitrag der Integration unterschiedlicher Facetten sozialer Präsenz und deren Übertragung auf virtuelle Teams. Neben drei technologiebasierten Dimensionen (1a Wahrnehmung anderer Teammitglieder als reale Person, 1b Wahrnehmung der eigenen Person als realen Teil des virtuellen Raums und 1c Unmittelbarkeit) wurden vier beziehungsbasierte Dimensionen (2a Vertrautheit, 2b Teamkohäsion, 2c sozialer Komfort und 2d gemeinsames Verständnis) berücksichtigt und ihr Auftreten in 148 erfolgreichen und nicht erfolgreichen Wissensaustauschsituationen getestet.

Eine erste, übergreifende Analyse zeigt, dass in 92 der berichteten Situationen soziale Präsenz nachgewiesen werden konnte. Darüber hinaus belegt die Verteilung der Auftretenshäufigkeit, dass die Präsenzkriterien wesentlich häufiger in erfolgreichen Wissensaustauschsituationen berichtet wurden (*n* = 68, 74 %) als in nicht erfolgreichen Situationen (*n* = 24, 26 %). Das stützt die angenommene Bedeutung für erfolgreichen Wissensaustausch.

Bezogen auf die Dimension der technologiebasierten sozialen Präsenz konnten zwei der drei Hypothesen (1a und 1b) anhand der Daten belegt werden. Die Annahme der *Hypothese 1a* zur *Wahrnehmung anderer Teammitglieder als reale Person *bestätigt die Bedeutung der bewussten und gegenständlichen Wahrnehmung des Gegenübers für erfolgreichen virtuellen Wissensaustausch. Die Kategorie wurde signifikant häufiger in erfolgreichen Wissensaustauschsituationen beschrieben und konnte zudem in den meisten kritischen Ereignissen (*n* = 48, 32 %) nachgewiesen werden. In virtuellen Settings ist die Wahrnehmung des Gegenübers stark von den technologischen Möglichkeiten und der Realitätsnähe des gewählten Mediums abhängig. In der menschlichen Kommunikation kommt jedoch besonders der Körpersprache eine herausragende Rolle zur Deutung von gesprochenen Inhalten zu (de Gelder und Hortensius 2014; Wang und Ruiz 2021). Mit der Bildübertragung wird die Mimik des Gegenübers sichtbar und ermöglicht ein zusätzliches Feedback zur verbalen Interaktion. Dies scheint besonders wichtig für den Erfolg des Wissensaustausches. Die häufige Nennung dieser Kategorie geht einher mit deren Priorisierung in vorangegangener Forschung, in der die Realitätswahrnehmung anderer Personen als zentrales Kriterium sozialer Präsenz betrachtet wird (Kreijns et al. 2014; Oh et al. 2018).

Die Bestätigung der zweiten *Hypothese 1b* zur *Wahrnehmung der eigenen Person als realen Teil des virtuellen Raums* belegt ebenfalls die Bedeutung der technologiebasierten Präsenzwahrnehmung für virtuellen Wissensaustausch. Das Gefühl vor Ort zu sein und sich als Teil des Teams wahrzunehmen ist zum größten Teil von den technologischen Gegebenheiten abhängig. Eine hohe Qualität der Bild- und Tonübertragung sowie der Zugang zu vielfältigen Kommunikationskanälen kann die physische Distanz in virtuellen Meetings ausgleichen (Karl et al. 2021).

Die dritte *Hypothese 1c *zur Rolle von *Unmittelbarkeit *der Interaktion für den Erfolg des Wissensaustausches konnte nicht bestätigt werden. Unmittelbarkeit wurde nur in 11 (7 %) kritischen Ereignissen nachgewiesen. Aufgrund der geringen Auftretenshäufigkeit scheint der Unmittelbarkeit insgesamt eine geringere Relevanz für Wissensaustausch in den untersuchten Teams zu zukommen. Dies deutet daraufhin, dass es wahrscheinlich von untergeordneter Bedeutung ist, ob der Wissensaustausch direkt oder indirekt stattfindet. Weitere Studien mit umfassenderen Datenerhebungen sind notwendig, um die Rolle der Unmittelbarkeit beim Wissensaustausch genauer zu analysieren.

Die Rolle der beziehungsbasierten Dimension sozialer Präsenz wurde mit den Hypothesen 2a, 2b, 2c und 2d getestet. Drei der vier Hypothesen (2a, 2b und 2c) konnten bestätigt werden. Das unterstreicht die Bedeutung dieser Dimension für die virtuelle Teamarbeit.

Mit Bestätigung der *Hypothese 2a* wurde ein positiver Effekt der *gegenseitigen Vertrautheit* auf den Wissensaustausch nachgewiesen. In erfolgreichen Wissensaustauschsituationen berichteten die Teilnehmenden signifikant häufiger, dass sich die Teammitglieder persönlich kennen und eine Beziehung zueinander besteht. Bisherige Befunde konnten bereits eine positive Wirkung sozialer Verbundenheit und gegenseitigen Vertrauens auf die Bereitschaft Wissen zu teilen nachweisen (Yoon und Rolland 2012). Die vorliegenden Ergebnisse zeigen darüber hinaus, dass sich wahrgenommene Vertrautheit positiv auf die Qualität und den Erfolg des Wissensaustausches auswirken kann.

Die Bestätigung der *Hypothese 2b *zur Rolle der *Teamkohäsion* belegt die Bedeutung des Teamzusammenhalts für virtuellen Wissensaustausch. In Präsenzteams stellt Teamkohäsion eine Grundvoraussetzung für das Gelingen von Wissensaustausch dar. Dieser Zusammenhang ist empirisch bestätigt (Kakar 2018; Reagans und McEvily 2003). Die vorliegende Studie weist daraufhin, dass das Zusammengehörigkeitsgefühls auch in virtuellen Teams eine wichtige Voraussetzung für Wissensaustausch ist. Dies lässt sich damit begründen, dass ein ausgeprägtes Zugehörigkeitsgefühl die Identifikation mit dem Team unterstützt und dazu beiträgt, Unsicherheiten und Risiken in technologievermittelten Interaktionen zu überwinden (Izmirli 2017). Dies ist eine relevante Grundlage für offene Kommunikation und Interaktion als Grundlagen des Wissensaustausches.

Ebenfalls bestätigt wurde die *Hypothese 2c *bezüglich der Rolle *sozialen Komforts*. So berichteten einige Teilnehmende, dass erfolgreiche Wissensaustauschsituationen geprägt waren von Spaß und einem offenen und vertrauensvollen Miteinander. Diese positive Atmosphäre fördert die Bereitschaft, sich im Team aktiv mit dem eigenen Wissen einzubringen. Dieses Ergebnis steht in Einklang mit Befunden zu verwandten Konzepten, wie bspw. Teamklima und Vertrauen. So konnten u. a. Xue et al. (2012) nachweisen, dass ein unterstützendes Teamklima einen positiven Effekt auf virtuellen Wissensaustausch hat. Gleiches belegen Eisenberg und Krishnan (2018) für eine vertrauensvolle Atmosphäre.

*Hypothese 2d* zur Rolle des *gemeinsamen Verständnisses* in virtuellen Teams für den Erfolg des Wissensaustausches (Hypothese 2c) konnte dagegen nicht bestätigt werden. Obwohl diese Kategorie häufiger in erfolgreichen Situationen (*n* = 20) als in nicht erfolgreichen Wissensaustausch (*n* = 8) nachgewiesen wurde, war der Unterschied in der Verteilung nicht signifikant. Dies steht im Gegensatz zu vorangegangenen Studien, die die Bedeutung eines gemeinsamen Verständnisses und geteilter mentaler Modelle für effektiven Wissensaustausch hervorheben (Hong und Vai 2008; Widjaja et al. 2017). Eine mögliche Erklärung für den negativen Befund liegt in den untersuchten Teamarbeitskontexten begründet. Die befragten Teammitglieder berichteten schwerpunktmäßig Erfahrungen in der virtuellen Projektarbeit im IT-Kontext. Im Vergleich zu internationalen Teams mit unterschiedlichem kulturellem Hintergrund, wie sie u. a. von Hong und Vai (2008) und Widjaja et al. (2017) untersucht wurden, spielt das gemeinsame Verständnis für diese Teams wahrscheinlich eine untergeordnete Rolle bezogen auf die Zusammenarbeit und den Erfolg des Wissensaustausches.

## Limitationen, Implikationen und Fazit

### Limitationen und Implikationen für die Forschung

Die vorliegende Untersuchung basiert auf der Methode der kritischen Ereignisse bzw. auf halbstrukturierten Interviews (Flanagan 1954). Die Verwendung eines standardisierten Kategoriensystems sichert die Objektivität. Dennoch ist die Generalisierbarkeit der Ergebnisse eingeschränkt. Ergänzend sollten somit die vorliegenden Ergebnisse mit einer größeren Stichprobe und standardisierten Erhebungsverfahren validiert werden.

Eine weitere Einschränkung der vorliegenden Untersuchung liegt in Art der Stichprobe begründet. Datenbasis bilden Erfahrungsberichte aus virtuellen Teams in der deutschen IT-Branche. In weiteren Studien ist die Übertragbarkeit der Befunde auf weitere Teamkontexte und Settings zu prüfen. Wie bereits in der Diskussion der Ergebnisse aufgezeigt, können Präsenzkriterien, wie bspw. ein gemeinsames Verständnis, in Abhängigkeit von den spezifischen Anforderungen und Rahmenbedingungen eines Teams, eine unterschiedlich starke Bedeutung für Wissensaustausch haben. Dies könnte die Ergebnisse beeinflussen.

Das Kategoriensystem für soziale Präsenz wurde im Rahmen der vorliegenden Untersuchung aus der Literatur erarbeitet. Die genutzten Kriterien bieten einen übergreifenden Rahmen, der vorangegangene theoretische und empirische Arbeiten integriert und im Kontext virtuellen Wissensaustausches untersucht. Künftig gilt es zu prüfen, ob sich diese Kategorien in anderen Kontexten bestätigen lassen. Auch die Beziehung einzelner Kriterien zueinander und die Stärke der Einflüsse auf den Erfolg virtueller Zusammenarbeit blieb bisher unbeachtet und könnte neue Erkenntnisse liefern. Dabei gilt es in erster Linie zu überprüfen, ob und wie stark eine direkte kausale Beziehung zwischen den Kategorien der sozialen Präsenz und dem Wissensaustauscherfolg nachweisbar ist. Dafür eignen sich beispielsweise experimentelle Feldstudien. Es scheint ebenfalls aussichtsreich, das gemeinsame Auftreten und mögliche Wechselwirkungen zwischen den einzelnen Präsenzkategorien zu analysieren. Daraus könnten sich Rückschlüsse auf die Häufigkeit des gemeinsamen Auftretens verschiedener Kriterien ergeben und eine gegenseitige Einflussnahme wäre prüfbar.

### Implikationen für die Praxis

Auch in der Praxis ist das Gelingen des Wissensaustausches eine wichtige Voraussetzung für den Erfolg virtueller Teamarbeit (Hung et al. 2021; Shah-Nelson et al. 2020). Aus den vorliegenden Befunden zur Rolle technologie- und beziehungsbasierter sozialer Präsenz lassen sich konkrete praktische Maßnahmen zur Unterstützung wissensintensiver Prozesse ableiten. Eine Möglichkeit ist, für eine hohe Realitätswahrnehmung zu sorgen, indem gegenseitige Vertrautheit, Teamkohäsion und sozialer Komfort geschaffen werden.

Bezogen auf die technologiebasierte soziale Präsenz ist insbesondere die Realitätswahrnehmung anderer und der eigenen Person durch den gezielten Einsatz reichhaltiger Medien zu fördern. Dazu eignen sich lebendige und realitätsnahe Formen von Kommunikationsmodalitäten, wie bspw. der Einsatz von Avataren. Diese fördern das Gefühl real anwesend zu sein und sich selbst als Teil des Wissensaustausches wahrzunehmen (Oh et al. 2018). Eine besondere Bedeutung für die wahrgenommene soziale Präsenz spielt die visuelle Darstellung der Kommunikationspartner und Teammitglieder. Durch die Verwendung von Videokonferenzen mit gegenseitiger Bildübertragung können nonverbale Signale der Teammitglieder gedeutet und unmittelbares Feedback bei Verständnisproblemen gegeben werden (Karl et al. 2021; Blanchard 2021). Dagegen hat eine fehlende Bildübertragung negative Auswirkungen auf das Erleben der Teamzugehörigkeit und verhindert, dass die Teilnehmer ihre Konzentration auf den Austausch legen.

Für eine hohe beziehungsbasierte soziale Präsenz sind gegenseitige Vertrautheit, Kohäsion und sozialer Komfort im Team zu unterstützen. Das heißt, es sollte nicht nur Präsenz im Sinne des „Da-Seins“ im Vordergrund stehen, sondern das „Gemeinsam-Da-Sein“ (Altschuller und Benbunan-Fich 2010) gefördert werden. So können bspw. gegenseitige Vertrautheit und Zusammenhalt gestärkt werden, indem Teammitgliedern die Möglichkeit geboten wird, sich regelmäßig persönlich und informell auszutauschen (Blanchard 2021; Gupta und Govindarajan 2000). Führungskräfte und Moderatoren sind angehalten, ihr Team zu einer kontinuierlichen Kommunikation zu motivieren. Das steigert die Motivation und fördert gegenseitiges Vertrauen (Zeuge et al. 2020). Eine wichtige Aufgabe der Führungskraft ist es somit, die Beziehungen der Teammitglieder zu stärken. Insbesondere da es virtuellen Teams an informellen, spontanen Kontaktmöglichkeiten mangelt (Leslie et al. 2018). Dazu eignen sich u. a. virtuelle Kaffeepausen, die Raum für informelle, spontane Gespräche geben. Virtuelle Führungskräfte können ebenfalls „Care Calls“ machen, um die Teammitglieder persönlich kennenzulernen (Zeuge et al. 2020). Durch den Aufbau einer zwischenmenschlichen Beziehung zwischen den Mitgliedern wird das Gefühl der Zugehörigkeit, Identifikation und persönlichen Präsenz im Team gestärkt (Shaik und Makhecha 2019). Weitere unterstützende Maßnahmen, wie ergänzende Face-to-Face-Meetings, eine hohe Meetingfrequenz und die konsequente Initiierung von Austauschsituationen, werden für den Aufbau einer vertrauensvollen Atmosphäre in virtuellen Teams diskutiert (Brahm und Kunze 2012; Coutu 1998; Iacono und Weisband 1997; Suchan und Hayzak 2001) und fördern das Erleben eines „Gemeinsam-Da-Seins“ im Team.

### Fazit

In der vorliegenden Untersuchung wurde erstmals die Rolle sozialer Präsenz für den Erfolg von Wissensaustausch in virtuellen Teams untersucht. Grundlage dafür bilden die aus der Literatur abgeleiteten zentralen Kategorien sozialer Präsenz, deren Auftreten in 148 kritischen Wissensaustauschsituationen analysiert wurde. Das verwendete Kategoriensystem erweitert bisherige Konzepte und Untersuchungen zum Einfluss sozialer Präsenz im Kontext virtueller Zusammenarbeit, indem neben technologiebasierte auch beziehungsbasierte Dimensionen sozialer Präsenz Berücksichtigung finden. Erstmals erfolgte die Analyse der Kategorien im Kontext von virtuellen Wissensaustauschsituationen. Die vorliegenden Ergebnisse weisen darauf hin, dass soziales Präsenzerleben für den Erfolg von Wissensaustausch in virtuellen Teams eine wichtige Rolle spielt. Durch gezielte Maßnahmen, wie die geeignete Wahl technologischer Medien, den Aufbau sozialer Beziehungen und die Unterstützung des Zusammenhalts und des Vertrauens in Teams, kann soziale Präsenz in virtuellen Settings gefördert werden.
